# Genetic Diversity and Population Structure in South African, French and Argentinian Angora Goats from Genome-Wide SNP Data

**DOI:** 10.1371/journal.pone.0154353

**Published:** 2016-05-12

**Authors:** Carina Visser, Simon F. Lashmar, Este Van Marle-Köster, Mario A. Poli, Daniel Allain

**Affiliations:** 1 Department of Animal and Wildlife Sciences, University of Pretoria, Pretoria, South Africa; 2 Instituto de Genética “Ewald Favret”, CICVyA-INTA, Hurlingham, Argentina; 3 INRA, UMR1388 GenPhySe, CS52627, Castanet Tolosan, France; Estonian Biocentre, ESTONIA

## Abstract

The Angora goat populations in Argentina (AR), France (FR) and South Africa (SA) have been kept geographically and genetically distinct. Due to country-specific selection and breeding strategies, there is a need to characterize the populations on a genetic level. In this study we analysed genetic variability of Angora goats from three distinct geographical regions using the standardized 50k Goat SNP Chip. A total of 104 goats (AR: 30; FR: 26; SA: 48) were genotyped. Heterozygosity values as well as inbreeding coefficients across all autosomes per population were calculated. Diversity, as measured by expected heterozygosity (H_E_) ranged from 0.371 in the SA population to 0.397 in the AR population. The SA goats were the only population with a positive average inbreeding coefficient value of 0.009. After merging the three datasets, standard QC and LD-pruning, 15 105 SNPs remained for further analyses. Principal component and clustering analyses were used to visualize individual relationships within and between populations. All SA Angora goats were separated from the others and formed a well-defined, unique cluster, while outliers were identified in the FR and AR breeds. Apparent admixture between the AR and FR populations was observed, while both these populations showed signs of having some common ancestry with the SA goats. LD averaged over adjacent loci within the three populations per chromosome were calculated. The highest LD values estimated across populations were observed in the shorter intervals across populations. The N_e_ for the Angora breed was estimated to be 149 animals ten generations ago indicating a declining trend. Results confirmed that geographic isolation and different selection strategies caused genetic distinctiveness between the populations.

## Introduction

The use of animal fibres from sheep and goats date back to the Biblical age, where wool was often used to make clothing, and goat hair for decoration in the temples [1]. The ancient Roman Empire facilitated the continued breeding of sheep and goats into the modern world, due in part to the continued importance of skins and hair in the making of clothing and water skins, as well as tents and stuffing for pillows [2].

The Angora goat originates from the district of Ankara in Turkey [1]. The Sultan of Turkey placed a ban on the export of both raw fleece and goats, and for several centuries they remained incarcerated in Turkey. The first records of exports date back to the late 18^th^ century in Spain and France and then later in the mid-19^th^ century, when a small number of goats were exported to both South Africa and the United States of America (USA). Similar to many other goat breeds, Angora goats have since spread throughout Europe, the Americas and Australasia via human-mediated distribution [3]. Other countries with notable mohair industries include Turkey, Lesotho, and Argentina [4].

During 1838 the first Angora goats were imported from Turkey to South Africa, with more than 3000 animals following these between 1856 and 1896. The harsh, semi-arid Eastern Cape region proved to be well-suited to the Angora goats, and a flourishing industry evolved from this beginning. Today the mohair industry in South Africa consist of approximately 700 000 Angora goats, producing in excess of 2 million kg mohair yearly [5]. The registration system for all seed stock animals in South Africa dates back to 1904 [6] and made provision for storage of pedigree information and official performance recording. Official performance recording for Angora goats was however only implemented in 1983, followed by the closure of the herd book in 1984 [7]. Due to restrictions on imports and exports, limited new genetic material has been available during the past five decades, and the SA gene pool has remained closed. Selection of Angora goats over the past four decades were primarily based on phenotypic selection. From the early 1970s until 1990, SA Angora goats have been intensely selected for increased mohair production. Body weight was compromised leading to smaller, unthrifty animals with an inability to survive sub-optimum conditions. Since then, animals have become larger but body weight is still of concern to breeders [8,9].

France started their mohair industry in the 1980’s, close to the time when mohair production approached a global decline. They maintained a small, but resilient niche market, and by 2006 had approximately 7500 pure-bred Angoras producing 30 tons of mohair annually [10]. Unlike most other countries where mohair is produced, the marketing of French mohair is a vertically integrated system, organised by farmers’ cooperatives [11]. Imports of genotypes from Texas (through Canada) and Australia up to the end of the 1980’s have formed the basis of the French Angora stock. A national selection scheme was developed in 1988 in collaboration with breeders, and since then no new imports were allowed in order to improve both quality and quantity of mohair produced by Angora goats. The selection scheme is driven by Capgenes (section Angora, Agropole, 2435 route de Chauvigny, 86550 Mignaloux Beauvoir, France), the National breeder organisation involving approximately 50 farmers. Objectives of selection are to increase fleece weight and to reduce both fibre diameter and undesirable fibre content. This selection scheme is based on an on-farm performance recording system, laboratory analyses for objective measuring of fibre diameter and undesirable fibre content, a national genetic database and the estimation of breeding values with a global index at national level.

In Argentina the Federal government imported some Angora and Tibetian goats in the early 19^th^ century, but the actual Angora population began with imported animals in 1962 from the USA. The goats were firstly located in the Northern part of the country but has since spread to the southern parts such as Patagonia. Additional animals were also imported from Australia and New Zealand [12]. Today mohair production is located on the northern area of Patagonia in the provinces of Neuquén, Río Negro and Chubut. After the Puyehue volcano eruption in 2011 the Angora goat population was reduced dramatically and today the total population is estimated at around 350,000–400,000 animals (personal communication, Taddeo H).

The Federal government implemented a Mohair project in the mid-1990s with the main focus to establish a dispersed open nucleus scheme, connecting commercial flocks with the INTA experimental flock. Furthermore a strategy of dissemination by controlled mating and artificial insemination was applied. The selection goal was to increase mohair production (fleece weight) and quality, mainly relative to fibre diameter (fineness) and medullated fibre proportion. It is expected that the program will provide organizational and operational structure to maintain the provision of males to commercial flocks [13].

The genetic improvement of Angora goats until recently was based on quantitative research and application of microsatellite markers for mainly population genetics [14]. The development of the Illumina goat SNP50 beadchip (Illumina, Inc. San Diego, CA 92122 USA) featuring 53 347 Single Nucleotide Polymorphism (SNP) probes in 2012 [15] opened new opportunities for genomic research in goats. Although no fibre-producing breeds were included in the development of the consortium chip, validation of the chip in the Angora breed indicated a relatively high level of polymorphic SNP [16,17]. Studies based on SNP technology have been limited to population structure and LD analyses in South African, French, Canadian and Australasian goats [18–22].

Due to country-specific selection and breeding strategies, a collaborative project was established to characterize the Angora goat populations of South Africa, France and Argentina. In this study we analysed genetic variability of Angora goats from three distinct geographical regions to assess the influence of genetic and geographical isolation as well as divergent selection patterns.

## Materials and Methods

### Study areas and sample collection

Angora goat populations from SA, France and Argentina were included in the study. A first population of forty eight goats’ samples were obtained in South Africa (SA) from the Small-stock Biobank (31°29’38”S and 25°1’2”E) at Grootfontein Agricultural Development Institute (GADI). These goats are all farmed in the Karoo region of South Africa with an average altitude of 1279 meters (m) above sea level. The area is known for its dry climate with approximately 350 mm rain per year and temperatures ranging from 6°C (average minimum) to 22°C (average maximum) (SA Weather bureau, 2012).

A second population of 26 goats was obtained in France (FR) from seven different farm members of the French Angora selection scheme. These farms were located in the Western part (“Bretagne” and “Pays de Loire” regions) of France and in the South close to Pyrenean Mountains. Goats were raised under semi-extensive conditions (pasture with supplementary feeding during pregnancy). Climate conditions are temperate with approximately 800–1000 mm rain per year and temperature ranging from 1–3°C (average minimum) to 21–25° (average maximum).

The third population of 30 goats was obtained in Argentina (AR) from the Angora goat experimental flock of INTA National Institute of Agricultural Technology (41°8´S and 71°8´E), located in the Pilcaniyeu, Rıo Negro province. Goats were raised extensively in a rangelands area of approximately 920 m altitude. The climate is semiarid with dry summers and cold winters. Annual precipitation is between 250 and 400 mm and the annual average temperature is 7.58°C [23].

All animals were selected within these populations to minimize relatedness between individuals. Sampling was done in each country with the required ethical approval. Ethics approval for the South African part of this study was obtained from the Ethics Committee of the Faculty of Natural and Agricultural Sciences at the University of Pretoria (EC130618-060 & EC104-13). The whole blood samples were collected from the DNA biobank at GADI. French DNA samples included in this study are stored at LABOGENA DNA. Sperm collection was performed on bucks by Capgenes, which obtained the authorization from DGAL (Direction Générale de l'ALimentation) FR CC 860. Sample collection was not performed specifically for this project in either South Africa or France–blood or DNA samples that were already available were used. Blood sample collection from Argentinian goats were approved by the Institutional Committee for Care and Use of Experimental Animals of the National Institute of Agricultural Technology (CICUAE-INTA) under protocol number 35/2010 and followed the guidelines described in the institutional manual. Blood samples were collected into 20ml EDTA tubes from the jugular vein under the supervision of qualified veterinarians. This study did not involve animals from any endangered or protected species/breeds.

### Genotyping & data pruning

DNA was extracted at the various institutes using the Qiagen DNeasy Blood and Tissue Kit® and AxyPrep Blood Genomic DNA Miniprep Kit® kits following manufacturers’ protocols. Genotyping was conducted at the ARC-Biotechnology Platform (Onderstepoort, 0110) in South Africa (SA population), Labogena DNA platform (Domaine de Vilvert, CS 80009, 78353 Jouy en Josas cedex) in France (FR population) and GeneSeek (Lincoln, NE 68504) in the USA (AR population) using the Illumina goat SNP50 Bead chip (Illumina, Inc. San Diego, CA 92122 USA). All genotype calls were extracted from the raw data using GenomeStudio (Illumina).

The three data sets were subjected to quality control (QC) separately to assess both sample quality and differences between SNP genotyping, followed by merging of the three datasets. Sample- and marker-based QC was performed using PLINK [24] software. Samples with more than 5% missing genotypic data (sample call rate<95%) were removed from further analysis, while SNPs were removed based on average marker call rate (<95%), minor allele frequency (MAF<0.05) and Hardy-Weinberg Equilibrium (HWE *p*-value<0.001). For the calculation of heterozygosity and individual inbreeding, SNPs in linkage disequilibrium (LD) equating to an *r*^*2*^ value of more than 0.2 were also removed using PLINK’s—indep-pairwise command (SNP window size: 50, SNPs shifted per step: 5, *r*^*2*^ threshold: 0.2). In PLINK, an EM algorithm [25] is used to calculate *r*^2^ values between all SNP pairs across all autosomes.

### Genetic diversity and population structure analysis

PLINK was used for the estimation of mean expected (H_E_) and observed (H_O_) heterozygosity, as well as relatedness and average individual inbreeding coefficients (F_IS_), which were calculated for LD-filtered mapped, autosomal SNPs within and across the different sub-population. Relatedness was calculated as the proportion identity-by-decent (IBD) between individual pairs, as indicated by PLINK’s PI_HAT value. Arlequin version 3.5.2 [26] was used to detect differentiation within and between populations by means of an analysis of molecular variance (AMOVA). Population differentiation, as F_ST_, was also calculated using Arlequin [26].

The data of the three populations were merged and QC was again performed on the one dataset. Individuals with a sample call rate of less than 95% and SNPs that had a call rate below 95%, MAF below 5% or violated HWE (P<0.001) were removed from further analysis. After QC, 46 510 SNPs remained, of which 45 244 were autosomal. LD-based pruning was performed to remove SNPs that were in linkage disequilibrium with one another using PLINK’s simple pairwise threshold model (command:—indep-pairwise 50 5 0.2, as described above). This removed 30 139 SNPs that exceeded an *r*^*2*^ threshold of 0.2, after which 15 105 SNPs remained for further analyses.

PGDSpider version 2.0.8.2 [27] was used to convert PLINK.MAP- and.PED files to Arlequin format. In Arlequin, 1000 permutations were used for the AMOVA. Principal component (PCA) and population structure analyses were performed for LD-filtered mapped, autosomal SNPs. GCTA version 1.24 (Genome-wide Complex Trait Analysis) [28] was used to construct a genetic relationship matrix, and subsequently to estimate eigenvalues and eigenvectors for the first three principal components (command:—pca 3). Using ADMIXTURE version 1.23 [29], a cross-validation (CV) procedure was followed in order to determine the optimal K-value for population structure analyses. After CV errors were estimated for each K-value, the K-value with the lowest CV error was chosen as optimal. Genesis version 0.2.3 [30] was then used to generate population structure bar plots.

### Linkage disequilibrium (LD)

LD, measured as *r*^*2*^, was calculated for post-QC SNPs with known genomic location of autosomal chromosomes, using PLINK software. The *r*^*2*^ parameter refers to the squared correlation coefficient (*r*) between two variables—in this case, between the alleles at two separate SNP loci [31]. Here *r*^2^ is defined by the following formula;

$$r^{2}{(p}_{a},p_{b},p_{ab})=(\frac{{{(p}_{ab}-p_{a}p_{b})}^{2}}{p_{a}(1-p_{a})p_{b}(1-p_{b})},$$

where p_ab_ represents the frequency of haplotypes consisting of allele *a* at the first locus and allele *b* at the second locus [32]. Lewontin’s *D’* refers to the normalized measure of *D*, which is a parameter that quantitatively describes allelic association [33]. Estimates of *D’* are sensitive to allele frequency, especially when one allele is rare, and is inflated for small sample sizes. The *r*^2^ measure was therefore focused on for further characterization of LD.

Pairwise *r*^*2*^ values were calculated for each chromosome in each sub-population, as well as across sub-populations, with the—r2 command, using for the most part default settings. The—ld-window and—ld-window-r2 commands’ values were both set so that correlations between all possible SNPs were tested and that there was no *r*^*2*^ threshold, in order to encapsulate all possible linkage interaction between SNPs per chromosome. For each chromosome, *r*^*2*^ values were then sorted by inter-SNP distance, binned into different inter-SNP intervals (0-10kb, 10-20kb, 20-40kb, 40-60kb, 60-100kb, 100-200kb, 200-500kb and 500kb-1Mb), and averaged across the afore-mentioned intervals to observe possible *r*^*2*^ patterns for increasing inter-SNP distances.

### Effective population size (N_e_)

Effective population size (N_e_) was estimated using the recently available software, SNeP version 1.1 [34]. SNeP estimates N_e_ from genome-wide linkage disequilibrium (LD), using the following formula suggested by Corbin and co-authors [35],

$$N_{T\;(t)}=\frac{1}{\left(4f(c_{t})\right)}\;(\frac{1}{E\;[{r^{2}}_{adj}|c_{t}]}-\alpha$$,

where *N*_*T* (*t*)_ represents the past effective population size estimated t generations ago, *c_t_* represents the recombination rate *t* generations ago in the past, *r*^2^_*adj*_ represents the linkage disequilibrium estimation adjusted for sampling bias and α represents a constant. The recombination rate was calculated using the following equation suggested by Sved [36],

$$f\;(c)=c\;[(1-c/2)/{\left(1-2\right)}^{2}]$$

PLINK input files for quality filtered, autosomal SNP data sets were used for N_e_ calculation. Minimum and maximum inter-SNP distances of 0 and 1000Mb, respectively, were used. The data sets for each sub-population, as well as the merged dataset, were grouped into 20 distance bins of 50kb each. N_e_ estimates were subsequently calculated from the *r*^*2*^ values obtained for the average distance of each distance bin.

## Results

A total of 101 samples remained in the merged data set following quality control. Three individuals were removed from the Argentinian population due to poor call rate (<95%). The call rates were high for all three populations genotyped varying from 0.981 for the Argentinian population to 0.996 for the South African goats. After marker-based quality control (Table 1) 15.7%, 12.4% and 11.8% of SNPs were removed for the SA, FR and AR populations, respectively. The number of SNPs excluded, based on the respective QC parameters, differed markedly between sub-populations. Most markers failed based on MAF and HWE in the SA population, while sample call rate was a major cause of loss in the AR population. The SA goats were the only population in which HWE played a major role in exclusion of SNPs. LD-pruning removed 30 139 SNPs with 15 105 SNPs remaining for downstream analyses.

**Table 1:** Marker-based quality control results.

Population	SNP call rate<95%	MAF<5%	Polymorphic loci (%)	HWE (p<0.001)	SNPs remaining (%)
**Argentina**	3359	3623	49 724 (93.2)	92	47 075 (88.2)
**France**	1417	6152	47 195 (88.5)	149	46 751 (87.6)
**South Africa**	785	6262	47 085 (88.3)	1757	44 957 (84.3)
**Merged**	**1825**	**2603**	**50 744 (95.1)**	**3389**	**46 510 (87.2)**

The average relatedness, based on the proportion IBD, between individual pairs was calculated as 7%, 8% and 8% for AR, FR and SA populations, respectively, and was lower than relatedness between third-degree relatives (12.5%). Relatedness between individual pairs across populations was insignificantly small and average PI-HAT values range from 0% between FR and SA populations, to 0.05% between AR and FR populations.

Diversity, as measured by H_E_ ranged from 0.371 in the SA population to 0.397 in the AR population. These values decreased slightly when the LD-pruned datasets were used, as opposed to when all available SNPs were used. Inbreeding coefficients (*f*) calculated for each individual based on the observed and expected heterozygosity values showed limited loss of heterozygosity. The animal with the highest individual value of *f* (0.202) was found in the SA population. Within breeds, the SA population was the only population with a positive average *f* value of 0.009 (Table 2). After LD-pruning, all populations showed negative inbreeding values.

**Table 2:** Summary statistics for the three separate Angora sub-populations and the merged Angora population.

Population	Average MAF*	Average *PH*_E_*	Average *PH*_O_*	Average *PH*_E_**	Average *PH*_O_**	Inbreeding coefficient (*f*)*	Inbreeding coefficient (*f*)**
**Argentina**	0.29	0.397	0.414	0.397	0.417	-0.047	-0.051
**France**	0.26	0.380	0.378	0.369	0.375	-0.003	-0.016
**South Africa**	0.25	0.371	0.365	0.364	0.365	0.009	-0.0029
**Merged**	**0.30**	**0.398**	**0.368**	**0.400**	**0.382**	**0.081**	**0.047**

* Calculated across polymorphic SNP (after QC)

** Calculated after LD-pruning

AMOVA indicated that almost 12% of the variation was accounted for by variation among the populations, while 87.9% of the variation was due to within individual variation (Table 3). Across all populations, a fixation index (F_ST_) value of 0.12 was estimated.

**Table 3:** Analysis of molecular variance (AMOVA).

Source of variation	Degrees of freedom	Sum of squares	Variance components	Percentage of variation
**Among populations**	2	164566.679	1147.11285	11.86
**Among individuals within populations**	98	837910.341	27.13444	0.28
**Within individuals**	101	858079.500	8495.83663	87.86
**Total**	**201**	**1860556.520**	**9670.08393**	**100**

**Fig 1:**
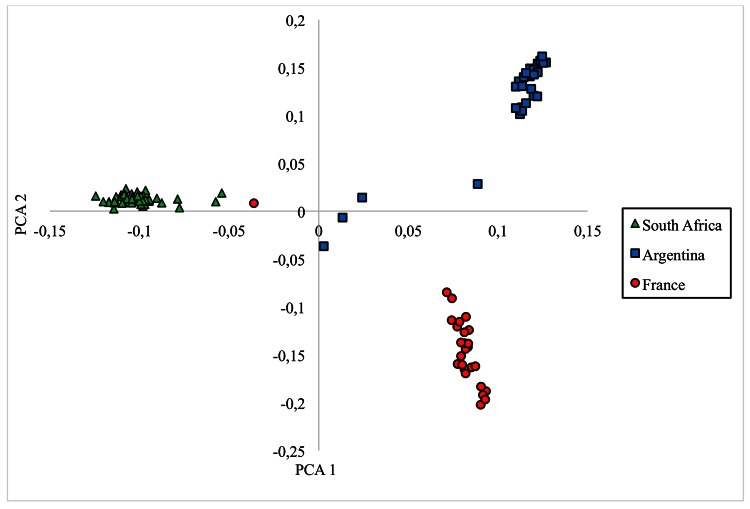
The genetic relationships among the 101 Angora goats as seen when plotting the first and second principal components (PCA1 and PCA2).

Principal component analysis (PCA) was used to visualize individual relationships within and between populations. The first, second and third principal components accounted for 47.3%, 35.3% and 17.4%, respectively, of the variability. All SA Angora goats formed a tight cluster, as shown in Fig 1. One outlier was observed for the FR population and four individuals for the AR population.

**Fig 2:**
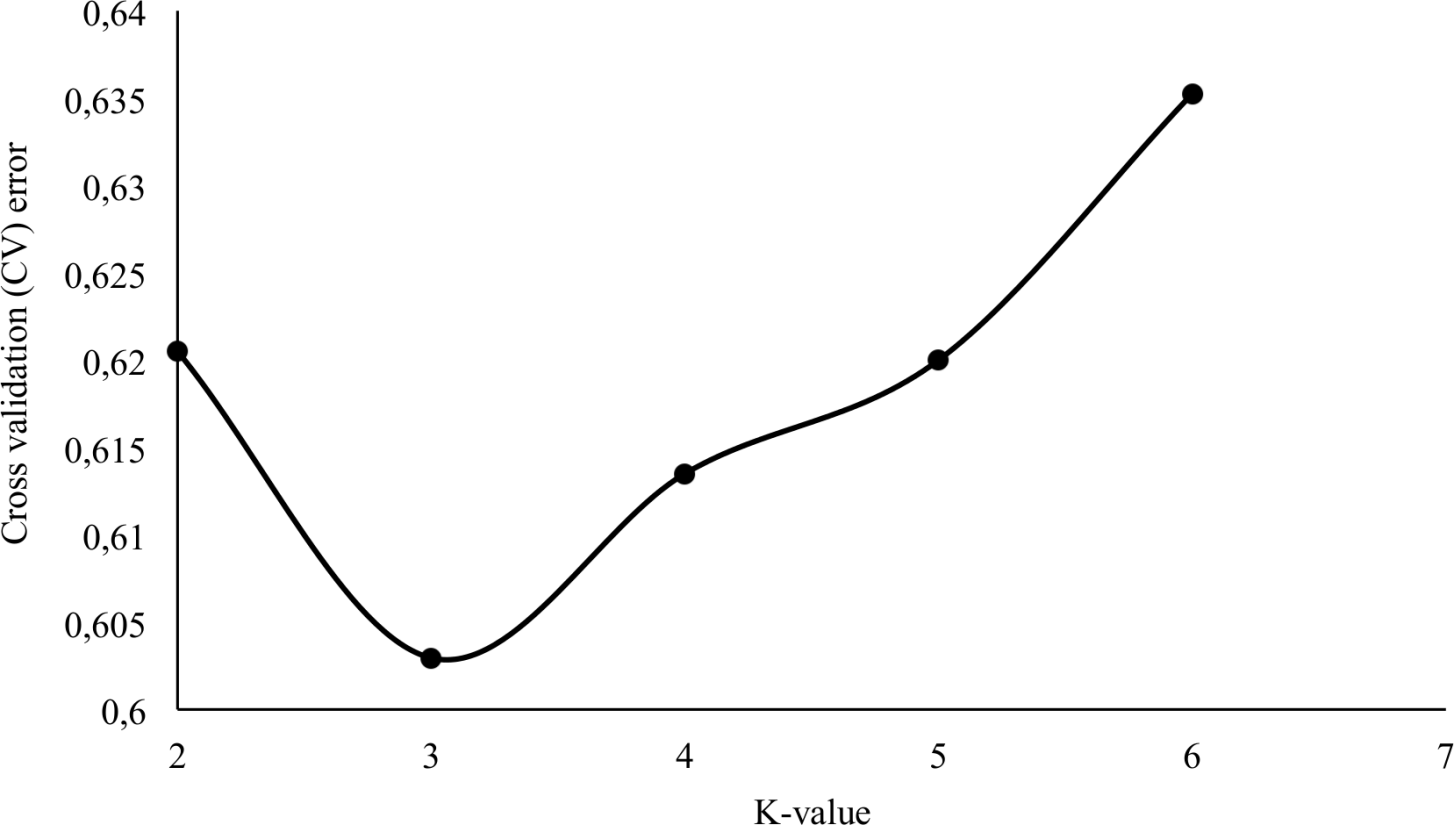
A cross-validation plot, indicating the choice of the appropriate K-value.

Likelihood scores for runs of various K-values in Admixture showed a decrease in cross-validation error values with an inflection point at K = 3 (Fig 2). At K = 2, the SA Angora goats already formed a separate cluster, while the AR and FR populations were grouped as one entity.

**Fig 3:**
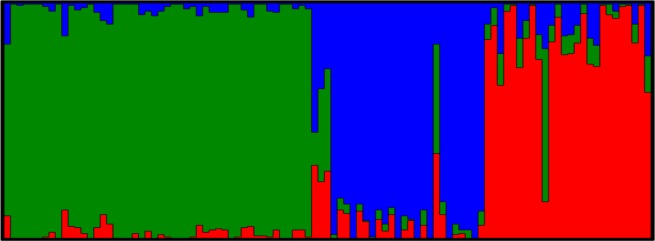
Population structure plot for K = 3 (Green: SA Angora, Blue: Argentinian Angora, Red: French Angora).

At K = 3 (as shown in Fig 3) geographic grouping is observed with the three populations’ individuals allocated into three distinct clusters. There is apparent admixture between the AR and FR populations, while both of them also show some common ancestry with the SA goats. Admixture plots for K = 2–4 have been added as supplimentary material and are displayed in S1 Fig.

In Table 4 the extent of LD averaged over adjacent loci within the three populations per chromosome are shown. *r*^*2*^ values across the 29 autosomes varied from 0.09 to 0.13, with an average of 0.11. The highest number of SNPs were observed on CHI 1 and the lowest on CHI 25.

**Table 4:** Linkage disequilibrium (LD) statistics per chromosome.

Chromosome	Number SNPs	D'	*r^2^*	Average distance (kb)	Min. distance (kb)	Max. distance (kb)
**CHI 1**	2921	0.47	0.11	53.04	1.82	280.97
**CHI 2**	2553	0.48	0.11	53.03	3.97	304.22
**CHI 3**	2134	0.46	0.11	54.73	8.16	306.69
**CHI 4**	2243	0.46	0.1	51.67	2.09	280
**CHI 5**	2025	0.47	0.11	54.79	3.68	431.32
**CHI 6**	2132	0.51	0.13	53.63	0.002	330
**CHI 7**	1995	0.49	0.12	53.43	4.51	333.29
**CHI 8**	2150	0.47	0.12	51.67	3.52	277.69
**CHI 9**	1735	0.45	0.1	51.98	6.31	236.99
**CHI 10**	1908	0.48	0.12	51.83	3.69	279.79
**CHI 11**	1963	0.48	0.12	53.56	3.38	260
**CHI 12**	1575	0.48	0.12	53.1	3.68	423.82
**CHI 13**	1509	0.48	0.11	53.44	0.03	393.85
**CHI 14**	1746	0.46	0.1	51.67	2.09	280
**CHI 15**	1494	0.47	0.11	52.89	2.46	434.35
**CHI 16**	1452	0.51	0.13	53.63	0.002	330
**CHI 17**	1363	0.46	0.1	52.62	3.57	475.02
**CHI 18**	1122	0.51	0.13	54.4	2.08	450.14
**CHI 19**	1099	0.47	0.11	56.37	7.34	276.3
**CHI 20**	1352	0.47	0.11	52.6	2.12	245.49
**CHI 21**	1276	0.47	0.11	52.24	1.09	237.6
**CHI 22**	1067	0.49	0.11	54.19	3.53	303.7
**CHI 23**	924	0.47	0.12	53.33	6.05	255.16
**CHI 24**	1206	0.48	0.11	50.99	3.67	327.87
**CHI 25**	757	0.45	0.09	54.69	4.46	750.33
**CHI 26**	964	0.44	0.11	51.96	6.11	394.97
**CHI 27**	852	0.45	0.11	51.68	5.78	382.71
**CHI 28**	839	0.45	0.09	51.27	1.04	258.99
**CHI 29**	880	0.43	0.09	54.97	3.46	241.85
**Average**		**0.47**	**0.11**	**53.08**	**3.44**	**337.35**

The highest LD values estimated across populations were observed in the 0-10kb and 10-20kb intervals (Table 5), followed by a gradual decrease. The AR population exhibited a lower LD across all intervals, compared to the SA and FR populations.

**Table 5:** Mean pairwise linkage disequilibrium (LD) estimates for different inter-SNP distance intervals.

Distance interval (kb)	Mean *r*^*2*^ ± SD			
	Argentina	France	South Africa	Total
**0–10**	0.35 ± 0.207	0.36 ± 0.221	0.38 ± 0.228	**0.32 ± 0.199**
**10–20**	0.20 ± 0.101	0.23 ± 0.119	0.23 ± 0.118	**0.17 ± 0.086**
**20–40**	0.16 ± 0.015	0.19 ± 0.020	0.19 ± 0.019	**0.13 ± 0.015**
**40–60**	0.14 ± 0.013	0.18 ± 0.015	0.18 ± 0.018	**0.11 ± 0.009**
**60–100**	0.13 ± 0.011	0.16 ± 0.015	0.16 ± 0.013	**0.09 ± 0.010**
**100–200**	0.11 ± 0.008	0.14 ± 0.014	0.14 ± 0.010	**0.08 ± 0.006**
**200–500**	0.10 ± 0.007	0.13 ± 0.013	0.13 ± 0.009	**0.07 ± 0.006**
**500-1Mb**	0.09 ± 0.007	0.12 ± 0.013	0.11 ± 0.008	**0.06 ± 0.005**

**Fig 4:**
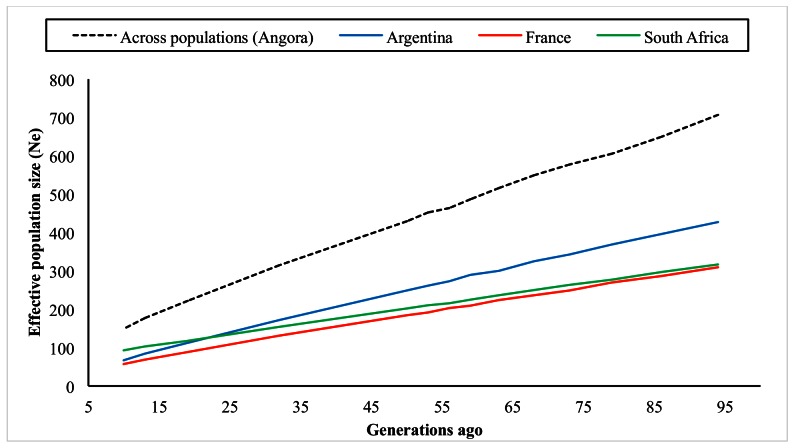
Trends in historic effective population size (N_e_).

**Fig 5:**
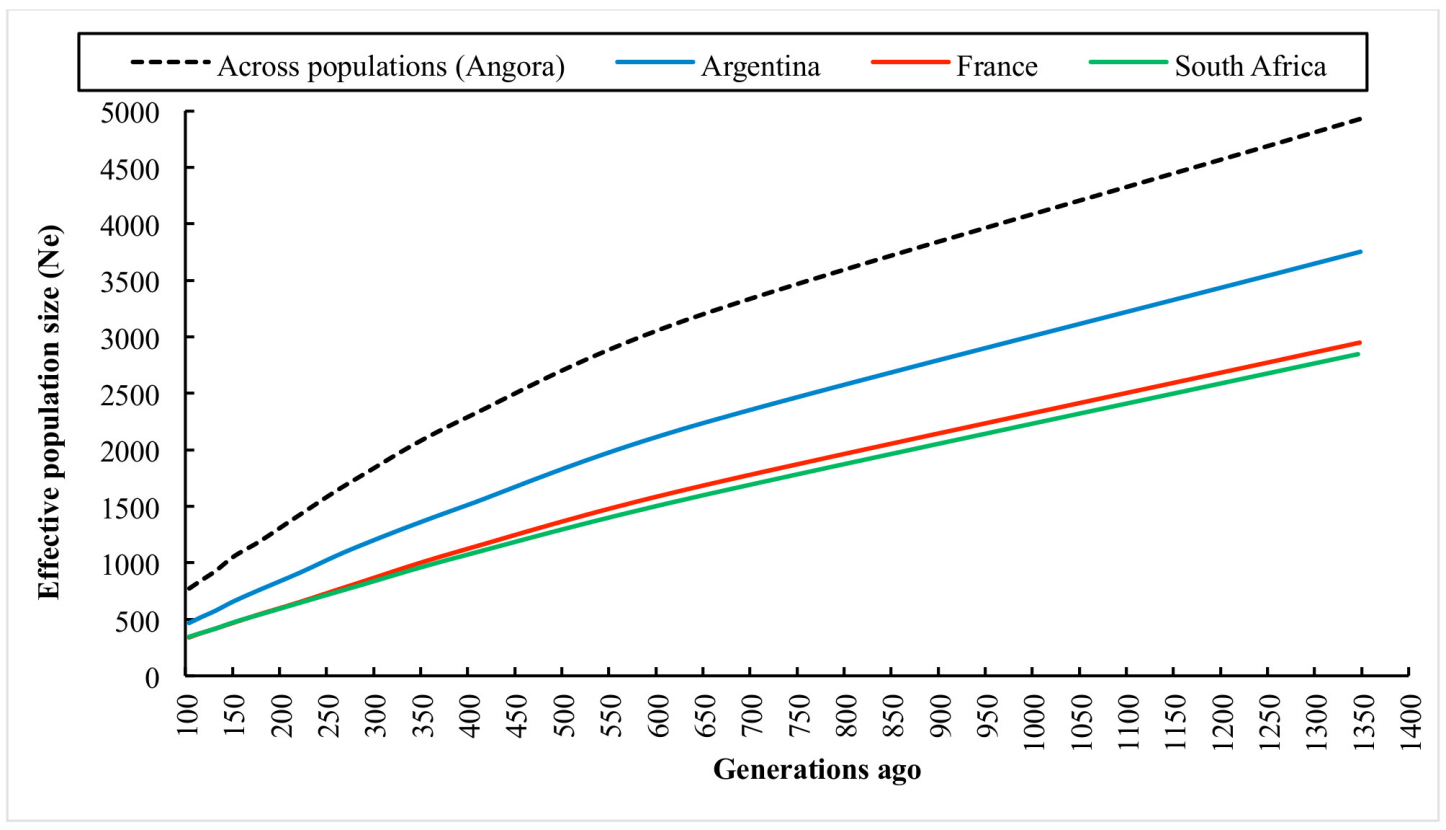
Trends in historic effective population size (N_e_) over 100 generations ago.

Figs 4 and 5 illustrate the trends in N_e_ up to 100 generations ago and more than 100 generations ago, respectively. All three populations show a marked decrease over time, as expected, but the current N_e_ is still of acceptable size with sufficient variation present in AR, FR and SA Angora goats. In the recent past, N_e_ was estimated to be 67, 57 and 93 for the AR, FR and SA subpopulations respectively, ten generations ago.

In the more distant past, N_e_ was estimated to be ~ 3750, 2950 and 2850 for the AR, FR and SA Angora sub-populations, respectively, at approximately 1350 generations ago. Limitations in the number of SNP pairs with short inter-SNP distances prevented the estimation of N_e_ past 1350 generations ago, and this is therefore the closest possible estimate to domestication, which occurred approximately 2500 generations ago [37].

## Discussion

Since domestication, livestock has been subjected to different forces of selection, including natural selection, genetic drift and directional selection for specific traits. These forces contributed to the genetic variation underlying phenotypic differences [38]. The Angora goat is no exception; subjected to these influences, sub-populations of this breed have been distinctly shaped in different geographical locations. Originating from Turkey, the Angora goat spread to various continents and were kept to a large extent in isolated populations. Over many decades the Angora populations were subjected to distinct breeding objectives and with the availability of modern genomic technology, the intuitive next step was to investigate the genetic diversity of the populations.

This study reports the results of three geographically-distinct Angora goat populations based on SNP technology. Marker-based quality control indicated that the SA population had the highest number of SNPs (1757) that did not adhere to HWE. This could be the result of strong directional selection in the SA population. The most polymorphic loci were typed in the AR population that also showed the highest H_E_ before and after LD pruning. This corresponds to the relatively large historic effective population size of the AR goats. The average H_E_ increased for both the FR and SA populations after LD pruning. The average H_E_ value over all populations (0.40) is comparable to the 0.44 estimate reported by [21] for Angora goats. Although the inbreeding coefficient estimated for SA was positive, the low value indicates no deliberate inbreeding in this selected population.

The variation among the populations in this study was almost 12%, which was unexpectedly high as these animals are all from one breed. The fixation index (F_ST_) of 0.12 confirms the differentiation of the three sub-populations within the Angora goats studied. Most diversity studies include a number of different breeds and these intra-breed diversity values are comparable to the current study, e.g. 12% variation among West African cattle breeds [39], 7.8% among six South African cattle breeds [40] and 10% among 38 horse breeds and populations [41]. The three Angora goat populations thus display the same level of differentiation as commonly seen between breeds.

The Principal Component Analyses clearly indicated three distinct populations according to their geographical distribution. The SA population has been isolated since its inception due to import restrictions (including long distances, veterinary concerns, low success rate of embryo transplants, etc.). This, in combination with strict phenotypic selection has resulted in a well-defined cluster. Compared to the other two populations, the FR goats are relatively more dispersed within their cluster. The French Angora goat population has been subjected to less intensive selection with a relatively young improvement scheme of less than 30 years. In the FR and AR populations, clear outliers were observed. The French outlier was identified as the only animal of the French sample without full pedigree information, and that was not the offspring of a Texan (via Canada) import. It is assumed that this animal could be the descendant of an Australian import (known to be connected to SA through Zimbabwean exports). This would also explain the high level of admixture in this individual. The four outliers in the AR population were also of Australian origin (at least three of them with one generation of pedigree information and most likely with SA links) as opposed to the other animals that were descendants from American imports.

The Structure analyses indicates a higher level of admixture between the AR and FR populations, most likely due to their original importations from the USA and Australia. It is believed that some Angora goats in Australia originated from SA via bordering African countries. This explains the relatedness between the SA population and the other two groups. These results indicate that although the Angora goat as a breed has migrated across continents, the dispersion is relatively recent and different populations still show common ancestry.

LD is a useful tool as it describes the non-random association of alleles and loci due to selection, population history and breeding strategies [42, 43]. In this study the *r*^*2*^ value was used as a more informative parameter due to the small population size, rather than D’ [44, 45]. The LD for all populations was the highest for smaller distance intervals up to 20kb with a gradual decline thereafter. The AR population showed lower levels of LD across all size intervals than the other two populations, which could be attributed to its relatively larger N_e_ compared to them. The average *r*^*2*^ across chromosomes for all the populations was 0.11, which was similar to values of 0.14–0.17 reported by [20], but lower than some breeds (range: 0.14–0.29) reported by [19]. These results highlight the necessity for a denser goat SNP chip, especially for specialized breeds with small population sizes.

The N_e_ for the Angora breed, across the subpopulations sampled in this study, was estimated to be 149 animals ten generations ago and, considering the declining trend observed, is expected to be even lower today. This trend could pose a threat to the genetic diversity of the Angora goat population, and should be monitored routinely to ensure a continuous sustainable mohair industry.

## Conclusion

It is important to note that the high diversity between populations could be used to exchange genetic material between populations. In this way outcrossing can contribute to improving certain unfavourable characteristics of specific populations (e.g. poor reproductive efficiency or growth rates) while maintaining superior mohair qualities.

## Supporting Information

S1 Fig Admixture plots for K = 2–4 showing population structure of different Angora sub-populations. (TIF)
