# Supplementary figures and images for: Genetic Diversity and Population Structure in South African, French and Argentinian Angora Goats from Genome-Wide SNP Data
Source: PLoS One. 2016 May 12;11(5):e0154353. doi: 10.1371/journal.pone.0154353 (PMC4865245; doi:10.1371/journal.pone.0154353)

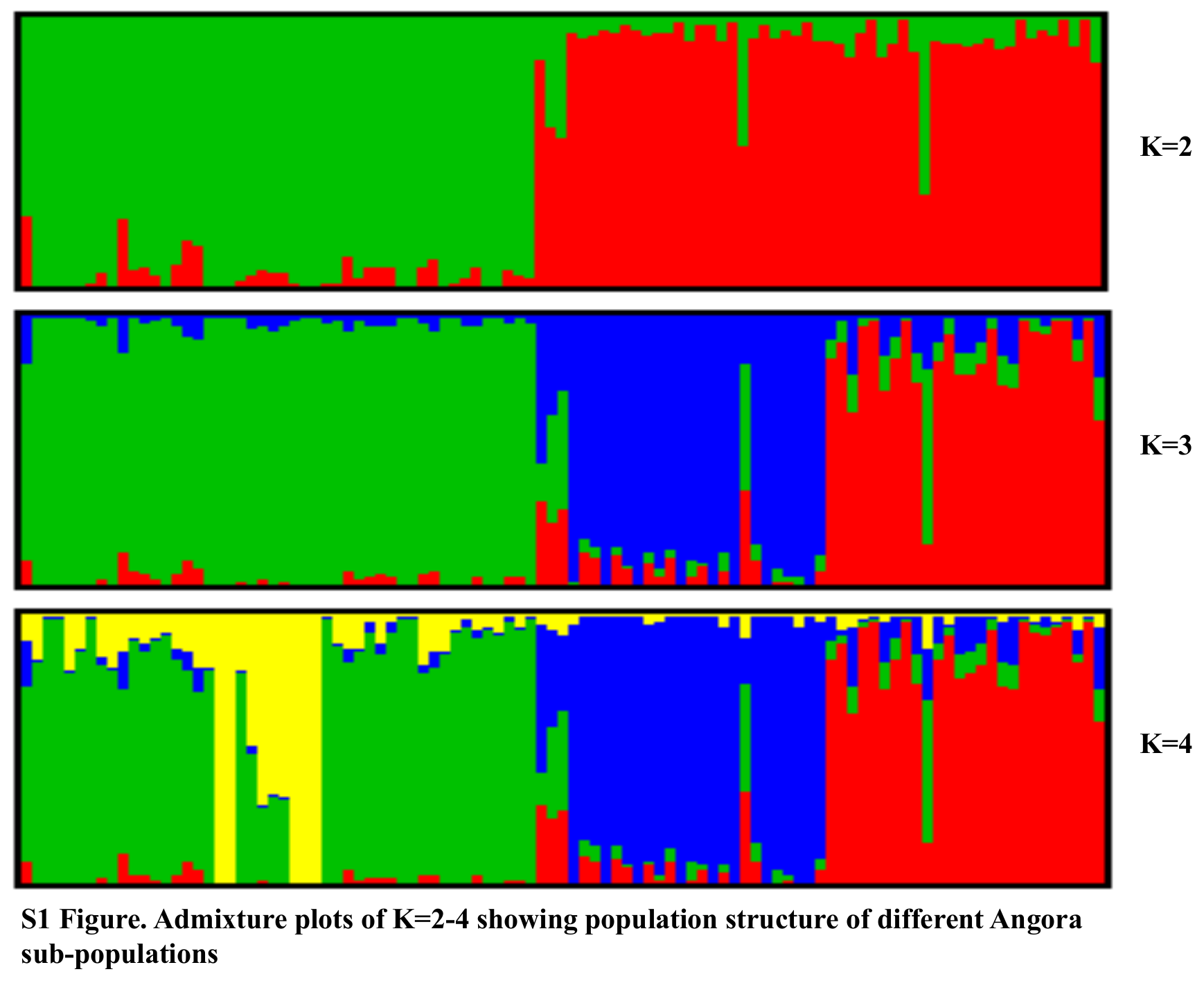

Supplement: S1 Fig — (TIF) [file pone.0154353.s001.tif]
